# Brexit and European doctors’ decisions to leave the United Kingdom: a qualitative analysis of free-text questionnaire comments

**DOI:** 10.1186/s12913-021-06201-0

**Published:** 2021-03-01

**Authors:** Adrienne Milner, Rebecca Nielsen, Emma Norris

**Affiliations:** 1 Division of Global Public Health, Department of Health Sciences, Brunel University London, Mary Seacole Building, 8 Kingston Ln, Uxbridge, UB8 3PN UK; 2 Barts and The London School of Medicine and Dentistry, Queen Mary University of London, Garrod Building, Turner St, Whitechapel, E1 2AD London, UK

**Keywords:** Brexit, Doctors, Migration, Identity, Personal and professional life

## Abstract

**Background:**

Quantitative evidence suggests that Brexit has had a severe and negative impact on European doctors, with many medical staff leaving the UK. This study provides a detailed examination of European doctors’ feelings towards Brexit, their intentions to leave the UK, and factors that may contribute to their potential decisions to migrate.

**Methods:**

An online questionnaire which included three optional free-text questions explored self-identifying UK-based, European doctors’ views of Brexit. The three questions prompted responses on how Brexit has impacted their personal lives, their professional lives, and their future migration decisions. Fifty-nine doctors participated in the questionnaire with 52 (88.1%) providing one or more responses to the three free-text questions. Twenty-seven doctors provided answers to all three free-text questions (51.9% of included sample). Thematic analysis was used to analyse this qualitative data.

**Results:**

Brexit was reported by the majority of participants to have a profound impact, although some respondents felt it was too soon to assess the potential consequences. Five themes emerged including: feeling unwelcome in the UK, Brexit as racism, uncertainty on legal ability to work, strain on relationships, and in contrast, a current lack of concern about Brexit.

**Conclusions:**

To mitigate the adverse personal and professional impact of Brexit, healthcare providers should provide financial and legal support to doctors applying for settlement in the UK, ensure they are addressing issues of racial and ethnic inequality in hiring, promotion, and pay, and work towards making clinical work environments inclusive for all staff and patients.

**Supplementary Information:**

The online version contains supplementary material available at 10.1186/s12913-021-06201-0.

## Background

The importance of healthcare workers and their ability to successfully carry out their work has been underscored by the COVID-19 pandemic. In the backdrop of COVID-19, Brexit remains an uncertainty in determining personal and professional satisfaction for European doctors working in the United Kingdom. Brexit was opposed by both The British Medical Association and Nursing and Midwifery Council [1, 2], with one study finding that 79.4% of doctors sampled voted to remain in the European Union (EU) in the 2016 referendum compared to 48.1% of general voters [3].

Although it is too early to conclusively state what the long-term impact of Brexit will be, Brexit has the potential to substantially affect the British health system. A post-Brexit economic downturn combined with a broad post-COVID recession has the ability to negatively impact the health system through multiple mechanisms [4]. For example, a devalued pound and imposition of trade barriers could make medicine procurement more expensive and difficult resulting in the de-prioritisation of the United Kingdom (UK) market for the introduction of new medications and devices [4, 5]. There is also the possibility of reduced public expenditure, resulting in increased pressure on the British social care system, which would negatively affect the health system [6, 7]. It is also unclear if trade deals with countries outside of Europe, such as the US, will diverge from current standards which, if lowered, may pose a risk to public health [8]. Moreover, the UK is no longer a member of the European internal market for information sharing and this may hinder knowledge exchange on the health workforce such as fitness to practice medicine [9]. Overall, poor economic performance and related repercussions on the health system may make the UK a more unattractive place to work for healthcare professionals, including doctors, which both directly and indirectly impacts the health system as a whole [4, 10].

Aside from the potential for clinicians, and especially European clinicians, to be less attracted to working in the UK post-Brexit, there may also be legal barriers that affect their ability to practice. Currently, health workers’ protection from EU directives have been written into UK law, but the UK is theoretically able to change these standards, which if dissolved, could negatively impact staff wellbeing and patient safety [10]. Throughout Brexit negotiations there was seldom much enthusiasm on either side to continue the mutual recognition of medical qualifications [11], and although the UK has stated it will continue to recognise EU and European Economic Area (EEA) gained qualifications for the next 2 years, this has not been reciprocated by EU and EEA countries [4]. Once the UK no longer recognises these qualifications it may deter European healthcare workers from remaining or migrating to the UK. In the past, legal barriers have been a factor preventing migration of Indian doctors [10], so this in effect may be replicated as qualifications from the EU and EEA will no longer be automatically valid in the UK beginning in January 2023.

There has been some indication from immigration statistics, academic research and anecdotal evidence that European staff are leaving the UK due to Brexit and other professional environment issues such as work pressure and understaffing, to which Brexit may have contributed [12–15]. In terms of understaffing, although it is difficult to pinpoint the level of vacancies in the National Health Service (NHS), it is estimated that between July and September 2018, there were nearly 94,000 full-time equivalent advertised vacancies in hospital and community services (including non-clinical roles) representing an 8% shortfall in posts [16]. Doctors and dentists represent 9217 of these vacant posts with the majority of vacancies being for nurses and midwifery at 37,917 [16]. Not only do these statistics show difficulty in recruiting and retaining staff, but also demonstrate challenges in terms of workload and associated stress for doctors currently practicing in the NHS, with these challenges even more amplified by the COVID-19 pandemic. It is recognised that stress at work is one reason for doctors quitting medicine [14, 17] and many are already quitting – a troubling sign for the NHS that Brexit may exacerbate staffing losses [7, 13, 18].

### Brexit: personal and professional life

A poor clinical working environment has negative consequences for staff mental health and well-being [19, 20], quality of care [21] and patient safety and outcomes [22, 23]. Because 10% of NHS doctors have qualified in EU or EEA countries [24], and other doctors may have qualified in the UK but hold European citizenship or are connected to Europe through family members or colleagues, there is potential for Brexit to significantly impact the entire health system. Since the referendum, there was a steep (89%) decline in EEA nurses entering the registry [25], as well as a 91% drop in EU and EEA applicants to join the Nursing and Midwifery register [26]. Though similar data showing quantitative outcomes post-referendum has not yet been collected for doctors, surveys carried out by the British Medical Association found that between 42 and 45% of European doctors were considering leaving after the referendum result and 18% had already made plans to leave [12, 13].

It is often difficult to disentangle personal and professional factors on determining migration decisions. For example, an Icelandic study [27] of migrating specialist doctors noted that personal financial reasons were an important determinant of migration decisions because finances were related to well-being and happiness; however, it can be argued that personal finances are impacted by employment factors such as location of employment and rank. This invites the question as to whether the same association could be applied to political circumstances which may affect doctors’ personal well-being and happiness. Indeed, it has been suggested that Brexit may have led to some degree of psychological distress from the uncertainty of immigration status, ability to work, feeling of national rejection and the economic implications of Brexit [28].

Other identified reasons relative to European doctors’ migration decisions post-referendum were the unanticipated concern about their rights and those of their families, as well as the potential loss of recognition of their qualifications [11, 29]. Indeed, it has been confirmed that mutual recognition of doctors’ qualifications will not continue now that the Brexit transition period has ended [30]. Previous research has shown that legal status or a change in such, may alter identity and feeling of belonging to a certain group or nationality [31].

#### Brexit, civic citizenship, and feelings of belonging

Changing legal status in addition to remaining uncertainties post-Brexit may threaten Europeans’ civic view of citizenship, in which citizens share a legal status and contribute and form part of the legal and financial framework of a nation [32]. Civic citizenship, as a component of national identity, contrasts to the cultural component of national identity which involves the feelings of shared culture, language and customs and ultimately a sense of belonging to a nation [31–33].

An additional consideration regarding European doctors’ feelings of belonging or lack thereof and their decisions to leave the UK relates to racism and xenophobia. The Leave campaign ran heavily on anti-immigration messages that consequently resulted in legitimising existing xenophobia and an increase in hate crime around the time of the referendum [34, 35]. Such sentiments, by both the population and politicians such as labelling Europeans as ‘queue jumpers’ [36] has created a sense of othering and hence threatens the cultural component of national identiy [12, 37, 38]. A sense of fitting in and belonging are important factors in determining happiness after migration, and may form part of the decision for migrants to return to their country of origin [33].

From the literature it is evident that the decision for doctors and other healthcare staff to migrate is multi-factorial. Yet, this research rarely considers migration factors potentially unique to Brexit such as sudden feelings of rejection and xenophobia. Though there has been quantitative evidence that Brexit has severely impacted European doctors and many medical staff have already left the UK, this study aims to conduct a more detailed examination of European doctors’ feelings towards Brexit, their intentions to leave the UK, and factors that may contribute to their potential decisions to migrate. Apart from one interview-based study of EU doctors working at two NHS England trusts [39], to our knowledge, this is the first study to collect qualitative data on Brexit and migration intentions from European-identifying doctors in the United Kingdom.

## Methods

### Design

A cross-sectional online questionnaire (suppletory file 1) explored European doctors’ views of Brexit. Three optional free-text questions within this questionnaire allowed participants to express their views in more detail.

### Participants

The online questionnaire was open to European doctors working in the UK. A European doctor in the UK was defined as a doctor who self-identifies as being from an EU, EEA, Swiss or EU candidate country, through legal nationality or other connections. Fifty-nine doctors self-identifying as being European doctors participated in the questionnaire. Fifty-two (88.1%) of the European doctors provided one or more responses to the three qualitative questions provided, with these presented in this study. Twenty-seven doctors provided answers to all three qualitative questions (51.9% of included sample).

### Instrumentation

Open-ended questions within a larger online questionnaire were assessed in this study. The overall questionnaire included 24 questions taking around 10 min to complete. Quantitative analysis of national identity assessment and perceived effects of Brexit on personal, professional life and future plans are assessed in a companion paper.

Twenty questions assessed various demographics of respondents, including gender, age, ethnicity, nationality, number of years living in UK, settled status in the UK, training grade, number of children, current relationship status, position on Brexit in 2016 (time of the Brexit vote) and their current position on Brexit. Perceived national, British and European identities were also assessed on a scale from 0 to 100 with 100 representing a very strong identification with their home nationality, Britain or Europe.

Perceived impact of Brexit was assessed with three follow-on open-ended questions. Firstly, ‘*On a scale of 0-100, with 0 being no impact and 100 being very much impact, please circle how Brexit has affected your personal life or not? Please explain why or why not*’ (*n* = 49/52 of participants providing any qualitative data; 94.2%). Second, ‘*On a scale of 0-100, with 0 being no impact and 100 being very much impact, please circle how Brexit affected your professional life or not? Please explain why or why not’* (*n* = 45/52 of participants providing any qualitative data; 86.5%). Finally, *‘In terms of your future plans, please circle which of the following best describes you? I am not considering leaving the UK; I am considering leaving the UK, but Brexit has not had any impact on that decision; I am considering leaving the UK, and Brexit has had an impact on this decision; I am leaving the UK, but Brexit has not had any impact on this decision; I am leaving the UK, and Brexit has had an impact on this decision. Please explain why or why not’* (*n* = 31/52 of participants providing any qualitative data; 59.6%).

### Procedure

Snowball sampling was used to recruit European doctors in the UK [40], from March to April 2019. The questionnaire was set-up on SurveyMonkey and distributed for voluntary completion via Facebook groups; ‘The Political Mess’ and ‘The Consulting Room’ which serve as forums for medical and political issues for predominantly UK-Based Doctors. Personal contacts were also contacted to participate, with one author (AM) working in a medical school and another (RMWN) being a medical school student at the time of data collection. The study was advertised as looking for EU/EEA/Self-identifying European doctors in the UK. Informed consent was requested, with participants advised the purpose of the research. No incentives were offered for questionnaire completion. Ethical approval was granted from Queen Mary University of London Ethics Committee (QMREC2257a).

### Data analysis

Thematic analysis was used to analyse free-text responses provided on the online questionnaire [41]. Related quotes across the three free-text questions asked were clustered to provide raw themes. An inductive approach was used to allow themes to emerge directly from the data [42]. Statements were read and re-read by two researchers, with emerging themes noted before being clustered into related concepts [41]. The number of participants reporting each theme was recorded.

## Results

### General demographics

The 52 participants had an average age of 38.9 (SD = 9.61), with 61.5% identifying as female. Although doctors from across the UK were eligible to participate in the study, 92.3% of respondents reported they were based in England, perhaps due to the authors’ location being in England. Thirty-nine participants (75.0%) described themselves as being of Caucasian European ethnicity, 4 (7.7%) other European ethnicity, 3 (5.8%) mixed ethnicity, 1 participant each identifying as Black, Chinese and Arab (1.9%), with 3 participants abstaining. Participants were nationals of 18 European countries, including Germany (13.5%), the Netherlands (11.5%), Ireland (9.6%), Spain (9.6%) and Italy (9.6%), as well as dual-nationals of Britain (19.2%), Australia (3.8%) and Bermuda (1.9%). 1.9% had lived in the UK for less than 1 year, 5.8% for 1–2 years, 19.6% for 2–5 years, 17.3% for 5–10 years, 40.4% for 10–20 years and 25% for over 20 years, with 34.6% having settled status in the UK. 11.5% were Foundation doctors (trainees), 30.8% were Specialty registrars, 21.2% were General Practitioners (GPs), 34.6% were Consultants and 1.9% were retired. 51.9% of the sample were married, 30.8% were cohabiting or in a relationship, 13.5% were single and 3.8% did not disclose their relationships status. 44.2% had at least one child.

### Brexit-related demographics

96.1% reported having the position for Britain to Remain in the European Union at the vote in 2016, with 3.9% being uncertain. This sample hence features a greater proportion of Remain voters than the 79.4% of doctors sampled in a previous study [3]. At the time of questioning (Spring 2019), 92.3% thought Britain should remain in the EU, 3.8% thought Britain should leave the EU and 3.8% were uncertain. Brexit was reported to have affected participants’ personal life (M = 71.56/100; SD = 30.26; *n* = 52) and their professional life (M = 53.33/100; SD = 37.48; *n* = 52). 34.6% of participants were not considering leaving the UK, 5.8% were considering leaving the UK but Brexit had not had any impact on their decision, 48.1% were considering leaving the UK with Brexit having an impact on this decision, 11.5% reported planning to leave the UK with Brexit having an impact on their decision and 0% reported planning to leave the UK but Brexit had not had any impact on their decision.

### Qualitative analysis of free-text questionnaire comments

#### Feeling unwelcome in the UK

The most commonly expressed perspective across the sample was no longer feeling welcome in the UK (*n* = 20/52; 38.5%): “*Got the message- I am no longer welcome here*”. This included description of a change in mentality within the British over time against Europeans: “*UK was open, friendly country when we moved here, part of Europe. We decided to stay because of tolerance and openness. This has changed and there is open hostility towards otherness*.” Participants described feelings of rejection from the UK as a country where they had built their lives: “*I am not welcome in a country that I love*”.

The Brexit vote was also described to shift doctors’ perceptions of their status in society towards an emphasis on their immigration status: “*I felt like an immigrant for the first time in 15 years*” and “*… There seems to be no appreciation for me as a doctor …*” . Different experiences were also evident depending on voting status of areas doctors were practicing in: *“I left a consultant job because it was in an area that voted leave. They didn’t want me there. Very demoralising”.* Some doctors (*n* = 6/52; 11.5%) described the Brexit vote and its aftermath as having clear effects on their mental health. They described increases in stress: *“Psychological stress, constant feeling of uncertainty*”, as well as specific stress to their private lives: “*this is bringing huge amounts of stress to my personal life*”.

#### Brexit as racism

Doctors (*n* = 7/52; 13.5%) explicitly described Brexit as indicative of racism within the UK where Brexit was perceived to reflect racist ideology. Brexit was described as an outlet for the British public to seek to promote their superiority over other countries and cultures: “*Brexit is essentially a racist ideology. It’s based on the uniquely special attributes of the British, their special qualities, their betterness then wretched foreigners”.* One doctor reflected on how their intersectionality was being betrayed by the British public in the aftermath of the Brexit vote: “*European Immigrant Female Doctors is probably the second worst thing to be now a days (right after maybe being a terrorist)”.* This reflects a demonization of ‘other’ groups portrayed by ‘Leave’ campaigners throughout Brexit, as well as a conflation of immigration status to terrorism. Anticipated future maltreatment of the British public by continuing to reside in the UK was also described: “*I do not want to live in fear of abuse and expulsion*”. One Caucasian female doctor shared an experience of overt, public racism shortly after the Brexit vote: *“In Nov 2016 I was subject to racist and sexist far right abuse with someone shouting Sieg Heil in my face and making the Hitler salute posture with his right arm”.*

#### Uncertainty on legal ability to work

Some doctors (*n* = 9/49; 18.4%) described their future working life in the UK to be insecure and uncertain. Three doctors described having felt forced to obtain British citizenship to secure their future in their UK, such as *“… in order to make sure I could live with my British children I had to swallow my pride, deny my belief [in a republic] and become a subject*” and “*made me take British citizenship*”. Some doctors also reflected that the UK leaving the EU may negatively impact their ability to return to secure work in Europe: *“If the NHS is not considered European, the years worked in the NHS will not count in case of future job applications in European countries”.*

#### Strain on relationships

A common theme (*n* = 11/52; 21.2%) was the strain that the Brexit vote and its’ aftermath had taken on doctors’ relationships. Working relationships were described to be under pressure in interactions between colleagues: *“The department is divided (doctors are remain and nurses leave), it polluted every human relationship in the hospital, all seen through the toxic prism of Brexit”.* Doctors also described the loss of high-quality European colleagues as they returned to other countries following the Brexit vote: *“Some of the best doctors and nurses I know have left the UK”.* Doctors’ research practice was also described to have been negatively affected by the UK’s changing Brexit status: *“Have had a research collaboration fall through.”* Patients were also described to make more unwanted inquisitions into doctors’ nationality and plans after Brexit: “S*uddenly the daily” *so where are you from*“ I get from patients seem to take a very british judgmental turn”.* For some doctors, these enquiries were too personal and unnecessary within the dynamic of doctor-patient interactions: “*Getting remarks and questions about my nationality and what my plans are after Brexit. It is annoying as it is a personal matter*”.

Personal relationships were also described to be affected by Brexit, with doctors describing uncomfortable dynamics within friendship groups: *“Some of my friends supported Brexit. This made me feel very uncomfortable”.* Importantly, extra demands at work seen as resulting from Brexit-related staff departures and supply delays were seen to bring difficulties in participants home lives. For example, one white Caucasian female commented: *“Extra workload impacts on longer days spent away from family … this has pushed me now to realise my health and that of my families health is more important”.*

#### Current lack of concern about Brexit

In contrast, eight participants (*n* = 8/52; 15.4%) described themselves as not being concerned on the effects of Brexit on their lives: “*nothing will change in my life*”. A common theme was that as Brexit was still ongoing at the point of questioning (Spring 2019), it was too early to know the effects on their lives: “*Brexit has not happened yet and nobody knows if there will be deal/no deal/part deal etc*”. For some, the lack of concrete post-Brexit plans encouraged them to postpone their worry: “*I will see what is it is like after Brexit actually happens!!”* and *“Too early to say. It may affect the conditions in which I have to work due to lack of personnel / resources.”* One Foundation doctor (trainee) described any changes from Brexit as being “*Unlikely to affect ability to finish training*”. One doctor commented that their lives would not be disrupted by additional elements of bureaucracy caused by Brexit: “*Filling out a form is not a big deal. I do this for the GMC, BMA, MPS, my hospital, the council, and HMRC on a regular basis. Whats one more for the government?*”

## Discussion

Our results show that Brexit was reported to have a profound impact on the majority of European-identifying doctors in our sample, although some participants felt it was too soon to know how Brexit may impact their lives. Five themes emerged from our participants including: feeling unwelcome in the UK, Brexit as racism, uncertainty on legal ability to work, strain on relationships, and in contrast, a current lack of concern about Brexit. This is consistent with the one previous known qualitative study [39] which found that Brexit made EU doctors feel unwanted and undervalued. However, whereas most participants in that study felt that Brexit would not affect their jobs or rights, respondents in our sample were generally more uncertain about their professional and legal future. This divergence in findings may be related to sampling where participants from the previous study only included doctors working at two NHS England trusts, who either had citizenship or had received their primary medical qualification from a member state of the European Economic Area.

Our results also show support for previous research suggesting that Brexit may result in psychological distress [28], feeling of national rejection and loss of nationality [28, 31], and fear of losing the recognition of qualifications earned elsewhere [31, 32]. Our findings related to perceived strain on working relationships are especially concerning as doctors continue to address the COVID-19 pandemic, because a poor clinical working environment has not only been linked to negative mental health and well-being consequences for staff [19, 20], but also quality of care [21] and patient safety and outcomes [22, 23]. Although our study did not pose specific questions on racism, we found that respondents discussed the link between Brexit and racism, unprompted by us, suggesting that further research in this area is necessary. Future research should also examine how Brexit is perceived by other health professionals apart from doctors as well as migrant health workers from regions outside of the EU/EEA. Because our study focused specifically on European doctors, this would enable a deeper understanding of how Brexit may affect the broader UK healthcare workforce.

It is clear from the participants in our study that there is anger, worry, and frustration, along with objective concerns about Brexit and legal status, qualifications, training and pensions, with this contributing to the strong impact of Brexit felt by participants in their personal and professional lives. Indirectly, many felt that Brexit had strained their work relationships, with the gross economic and staffing problems that could occur with Brexit negatively affecting the health of both patients and staff. As such, it is important that healthcare providers develop an understanding of Brexit’s impact on their clinicians and patients, as well as continually assess potential impact as the UK establishes its new relationship with Europe. From our results, it is evident that healthcare providers must demonstrate that European doctors are respected and appreciated in the UK in order to prevent further staff losses.

The use of free-text questionnaire data, although not as rich as interview data, provides a valuable method to elicit experiences and opinions quickly and at scale [43]. A strength of this study is the high proportion of optional free-text responses provided, with 88.1% of doctor participants providing responses to at least one of the three free-text questions. A limitation is that the majority of the sample was English, not representing experiences from other British nations. Another limitation is that because our inclusion criteria for self-identifying as “European” were broad, we are not able to quantify how many potential participants fit our study criteria as there are a number of ways UK-based doctors may identify as European (e.g., through citizenship, having a European parent, etc.). There may also have been self-selection bias of participants obtained by snowball sampling, whereby the vast majority of participating doctors voted Remain. These participants may have had stronger views about Brexit than the general population of doctors [3] and may have been more likely to respond to participant invitations. Furthermore, participants in our sample may also have had stronger concerns relative to how Brexit may impact their personal and professional lives. As such, self-selection into our sample may have resulted in increased attention devoted to the first four themes (feeling unwelcome in the UK, Brexit as racism, uncertainty on legal ability to work, strain on relationships) and decreased attention devoted to the last theme (a lack of concern about Brexit) evident in questionnaire responses than would have been observed if a different sampling method had been utilised. However, the goal of qualitative research is to represent the experiences of the included sample and not to generalise to larger populations [42]. As such we are not claiming that these results can be applied to all European-identifying doctors working in the UK.

## Conclusion

This study was carried out to examine how European doctors have felt Brexit has impacted their personal and professional lives and their intentions to leave the UK due to Brexit. To mitigate the negative personal and professional impact of Brexit, healthcare providers and the UK government should seek to take measures to ensure that European doctors feel valued and secure in their positions. Firstly, we welcome the decision to introduce the Health and Care Worker Visa and waiving the Immigration Health Surcharge for NHS workers. However, more steps could be taken to mitigate the effects of Brexit on the UK health workforce. The NHS and private providers could offer financial and legal support to doctors applying for settlement in the UK. Providers should also seek to alleviate European doctors’ feelings that they are not welcome in the UK and experiences of racism by ensuring they are actively addressing issues of racial and ethnic inequality in hiring, promotion, and pay and that clinical work environments are inclusive for all staff and patients. Furthermore, the UK government should seek to reduce fears surrounding legal ability to work for doctors specifically by targeting them to apply for the EU Settlement Scheme before the deadline on 30th June 2021. The government should also commit to retain current standards or strengthen workers’ rights and protections to ensure that the UK continues to be seen as an attractive place to work. Moreover, the EU and UK government should make additional arrangements to restore mutual recognition of qualifications to facilitate recruitment of doctors and other healthcare professionals to encourage potential new clinicians from European countries into the NHS. Although some of the damage of Brexit cannot be repaired, the UK medical system must focus on minimising its potential consequences.

## Supplementary Information

**Additional file 1.**

## Data Availability

The datasets generated and/or analysed during the current study are not publicly available because respondents did not give their informed consent that any anonymised data would be made available online. Data is available from the corresponding author on reasonable request.

## Abbreviations

EEA: European Economic Area
EU: European Union
GP: General practitioner
M: Mean
N: Sample size
NHS: National Health Service
SD: Standard deviation
UK: United Kingdom
