# Supplementary material for: Brexit and European doctors’ decisions to leave the United Kingdom: a qualitative analysis of free-text questionnaire comments
Source: BMC Health Serv Res. 2021 Mar 1;21:188. doi: 10.1186/s12913-021-06201-0 (PMC7917529; doi:10.1186/s12913-021-06201-0)
Supplement: Supplementary file 1 — Additional file 1. [file 12913_2021_6201_MOESM1_ESM.docx]

Supplementary file 1: Survey utilised

1) What is/are your legal nationality/nationalities?

2) Is there any other nationality, other than your legal nationality/nationalities, that you identify with? If yes, please state.

3) Aside from the UK, which countries have you lived in?

4) How would you describe your feeling of national identity on a scale of 0-100 with 0 being no sense of national identification and 100 being extremely strong sense of national identity?

5) How often do you visit the country of your nationality/nationalities?

6) How would you describe your feelings of British identity on a scale of 0-100 with 0 representing no identification with Britain and 100 representing a very strong identification with Britain?

7) How many years have you lived in the UK?

8) How would you describe your feelings of European identity on a scale of 0-100 with 0 representing no identification with Europe and 100 representing a very strong identification with Europe?

9) What was your position on Brexit at the time of the 2016 referendum?

- Leave

- Remain

- Uncertain

- No preference

10) What is your current position on Brexit?

- Leave

- Remain

- Uncertain

- No preference

11). Have you applied for settled status?

11a) If you have applied for settled status have you been successful?

12) On a scale of 0-100, with 0 being no impact and 100 being very much impact, please circle how Brexit has affected your personal life or not?

12a) Please explain why or why not

13) On a scale of 0-100, with 0 being no impact and 100 being very much impact, please circle how Brexit affected your professional life or not?

13a) Please explain why or why not

14) In terms of your future plans, please circle which of the following best describes you:

-I am not considering leaving the UK.

-I am considering leaving the UK, but Brexit has not had any impact on that decision.

-I am considering leaving the UK, and Brexit has had an impact on this decision.

-I am leaving the UK, but Brexit has not had any impact on this decision.

-I am leaving the UK, and Brexit has had an impact on this decision.

14a) Please explain why or why not

15) What is your race/ethnicity (i.e. Black, White, Hispanic, South Asian mixed race, other)?

16) What is your sex/gender (i.e. female; male, transgender, etc.)?

17) What is your age?

18) Please select your annual household income bracket:

- £0-24,999

- £25,000-49,999

- £50,000-74,999

- £75,000-99,999

- £100,000-149,999

- £150,000-199,999

- £200,000 or above

- Prefer not to say

20) In which postcode do you practice medicine?

21) What is your grade level? (i.e. FYI, CT, Register consultant, etc.)

22) What is your current relationship status (i.e., single, cohabitating, married, prefer not to say, etc.)

23) If you have a partner(s), what is their nationality/nationalities?

24) If any, how many children, do you have?
